# Skin color and mental health in Peru: the mediating role of experiences of discrimination among university students

**DOI:** 10.3389/fpsyg.2026.1867013

**Published:** 2026-09-30

**Authors:** Julio Dominguez-Vergara, Henry Santa-Cruz-Espinoza, Edmundo Salas-Salas

**Affiliations:** 1 Research Direction, Universidad Tecnológica del Perú, Lima, Peru; 2 School of Psychology, Faculty of Health Sciences, Universidad Autónoma del Perú, Lima, Peru

**Keywords:** colorism, discrimination, mental health, Peru, psychological distress, psychological well being, skin color, university students

## Abstract

**Objective:**

This study aimed to examine the mediating role of experiences of discrimination in the association between skin color and mental health among university students.

**Methods:**

A cross-sectional correlational design was used. The sample comprised 603 university students aged 18 to 38 years from Metropolitan Lima. Skin color was assessed using the PERLA color palette, experiences of discrimination were measured using the Experiences of Discrimination Scale, and mental health was assessed using the Mental Health Inventory-5 (MHI-5), considering its two-dimensional structure of psychological well-being and psychological distress. The analyses included descriptive statistics, Spearman’s rank correlations, linear regression models with HC3 robust standard errors, and mediation models with 95% bootstrap confidence intervals based on 5,000 resamples.

**Results:**

Darker skin tones were significantly associated with greater discrimination. In turn, discrimination was associated with lower levels of psychological well-being and greater psychological distress. No significant direct associations were found between skin color and either psychological well-being or psychological distress. The mediation analysis showed a significant indirect effect of skin color on psychological distress through experiences of discrimination, whereas the indirect effect on psychological well-being was not significant. Overall, the findings suggest that discrimination constitutes an important psychosocial pathway linking darker skin tones to greater psychological distress.

**Conclusion:**

In this sample, skin color was not directly associated with mental health; however, it acquired psychosocial relevance through its association with greater exposure to experiences of discrimination. These findings suggest that phenotypic hierarchies may be associated with the mental health of Peruvian university students through greater exposure to discrimination. Addressing discrimination within university settings may help reduce psychological distress and promote students’ mental health.

## Introduction

Ethnoracial inequalities are structured not only through categories of racial or ethnic identification but also through socially visible phenotypic characteristics shaped by skin-tone hierarchies. Although race and ethnicity generally encompass cultural, linguistic, and ancestral factors, skin tone is an observable phenotypic trait determined primarily by melanin levels and genetic factors (Mosca and Morrone, 2023; Naik and Farrukh, 2022). Within this framework, colorism is defined as differential treatment or discrimination based on skin tone (Patterson and Grob, 2026; Strmic-Pawl et al., 2021). Pigmentocracy, in turn, refers to a social order in which people with lighter skin tones tend to occupy positions of greater prestige and have greater access to resources, whereas those with darker skin tones experience greater social disadvantage (Telles et al., 2015; Woo-Mora, 2026). Accordingly, colorism and pigmentocracy are important mechanisms for understanding how phenotypic hierarchies contribute to social stratification and mental health inequalities (Harris, 2022; Painter II et al., 2020).

International evidence has documented the disproportionate effects of colorism across different social contexts and racialized communities. In English-speaking countries, this phenomenon has been widely studied, particularly among populations of African descent, showing that darker skin tones are generally associated with greater exposure to unequal treatment and social exclusion (Branigan and Hall, 2023; Dupree-Wilson, 2021; Oh et al., 2021). Similarly, studies conducted in the United Kingdom have documented experiences of colorism among both Black adolescents and adults from Black and Asian communities, suggesting that these phenotypic hierarchies extend across national contexts (Craddock et al., 2023, 2025). In Latin American societies, colorism is linked to racial hierarchies established during colonization and subsequently reconfigured through discourses of mestizaje and whitening (Charles, 2021; Verdín, 2024). Although mestizaje is often portrayed as a symbol of racial integration and equality, it can also hinder recognition of the inequalities that continue to be organized around physical appearance (Abramo, 2022; Wade, 2017). Comparative evidence from the Project on Ethnicity and Race in Latin America showed that darker skin tones were consistently associated with lower educational attainment across eight countries in the region, even after accounting for ethnoracial identification and social background. Nevertheless, the magnitude of these inequalities varied across countries and social groups (Telles et al., 2015). In countries such as Brazil, Mexico, and Puerto Rico, darker-skinned individuals face heightened discrimination in areas such as education, employment, and healthcare (Landor et al., 2026; Patterson and Grob, 2026; Travassos et al., 2011), whereas associations between skin tone and health appear to be partially explained by socioeconomic position and experiences of discrimination (Perreira and Telles, 2014).

Evidence regarding skin tone and mental health has also yielded heterogeneous findings. Skin-tone hierarchies may affect mental health through processes such as personal devaluation, diminished self-esteem, stigma consciousness, and other forms of emotional harm (Dent et al., 2020; Louie, 2020). Consistent with this perspective, studies have found that people with darker skin tones are more likely to experience discrimination, psychological distress, or certain psychiatric disorders (McCleary-Gaddy and James, 2022; Oh et al., 2021). However, other studies indicate that these associations may vary according to ethnic identity, gender, age, and the specific mental health outcome examined. For example, Bijou and Colen (2022) found that the association between skin tone and mental disorders differed across ethnic groups and did not necessarily follow a linear pattern in which darker skin tones were invariably associated with the most unfavorable outcomes. Similarly, Hamler et al. (2022) observed that skin tone moderated some associations between discrimination and mental health, but its effects were not consistent across all outcomes. Therefore, it is inappropriate to assume that skin pigmentation directly produces psychological consequences. Instead, the social processes through which skin tone may acquire relevance for mental health must be examined.

Experiences of discrimination represent one of the main pathways through which colorism may affect mental health. Discrimination constitutes a social stressor that can generate negative emotional responses, heighten vigilance toward possible threats, undermine feelings of safety and belonging, and require repeated coping efforts. Available evidence suggests that skin tone is associated not only with differences in mental health but also with greater exposure to unequal treatment, which may, in turn, increase emotional distress (Firat and Noels, 2022; Li et al., 2022; Uddin et al., 2022; Xiong et al., 2024). Meta-analytic evidence further indicates that discriminatory experiences are associated with immediate deterioration in mental health (Emmer et al., 2024), whereas intensive longitudinal studies have identified associations between ethnoracial discrimination and different mental health indicators among adolescents and young adults (Wang et al., 2026). Within the specific field of colorism, people with darker skin tones are more frequently exposed to unequal treatment, stereotypes, and appearance-related microaggressions, which may contribute to adverse psychological outcomes (Louie, 2020; Mercier et al., 2026; Monk, 2015).

Mental health is not limited to the presence or absence of psychological symptoms. The two-continua model proposes that psychological well-being and psychological distress are related but distinct dimensions. For example, a person may experience few psychological symptoms while simultaneously reporting low levels of well-being or may experience some degree of distress while retaining positive psychological resources (Mason Stephens et al., 2023; Westerhof and Keyes, 2010). Accordingly, psychological distress reflects the presence of symptoms such as anxiety, sadness, or emotional tension (Ohayashi and Yamada, 2012), whereas psychological well-being encompasses positive experiences that may also depend on protective resources, interpersonal relationships, and social conditions (Hako and Mbongo, 2024).

University education coincides with a period of psychosocial transition characterized by identity exploration, academic demands, and the need for social integration. Within this context, students from underrepresented groups face not only the stressors inherent to university life but also experiences of discrimination and exclusion, which may increase their psychological vulnerability (Freire and Hurd, 2023; Hua et al., 2023; Jochman et al., 2019). Longitudinal evidence further suggests that discrimination experienced during the early stages of university education may substantially undermine students’ sense of institutional belonging and, through this pathway, predict depressive symptoms and stress in subsequent years (Freire and Hurd, 2023). Studies of students attending predominantly White campuses have also shown that experiences of interpersonal discrimination are associated with higher levels of anger, anxiety, loneliness, and depressive symptoms, demonstrating the immediate emotional impact of these experiences on everyday university life (Jochman et al., 2019; Volpert-Esmond and Aboargob, 2023). Nevertheless, much of this evidence comes from universities in the United States. Therefore, these findings must be interpreted in light of the specific historical and social characteristics of the Peruvian context.

In Peru, ethnoracial inequalities have persisted over time despite discourses of mestizaje and continue to significantly affect Afro-Peruvian, Indigenous, and Amazonian populations (Abramo, 2022; Sánchez-Villena and Temple-Focón, 2025). These inequalities are expressed not only through explicit discriminatory acts but also through everyday practices, discourses, and institutional criteria that normalize differential treatment. In Mexico and Peru, racial humor targeting Indigenous and Afro-descendant populations may contribute to the reproduction of ideologies that minimize racism by portraying these expressions as harmless jokes (Sue and Golash-Boza, 2013). The coexistence of social recognition of racism and its denial at the individual level is reflected in the First National Survey of Perceptions and Attitudes toward Cultural Diversity and Ethnoracial Discrimination. In this survey, 53% of respondents considered Peruvians to be racist or very racist, whereas only 8% described themselves in those terms (Ministerio de Cultura, 2018).

Phenotype-related inequalities are also evident in access to educational opportunities. Among the Afro-Peruvian population, evidence suggests that phenotypic appearance, including skin color, influences educational opportunities and social mobility trajectories. Darker-skinned Afro-Peruvians are less likely to access higher education than their lighter-skinned counterparts (Benavides et al., 2019). These dynamics are also present in university settings, although they are not always fully recognized or discussed institutionally. For example, a study conducted at a public university in Lima, Peru, found that discussions of racism remained at an incipient stage and were primarily driven by Afro-Peruvian students. Through workshops and other initiatives, these students sought to break the historical silence surrounding this issue (da Silva, 2022). These studies demonstrate that skin tone may function as a marker of social position and differential exposure to particular experiences. However, they do not imply that all educational or psychological inequalities can be attributed exclusively to skin pigmentation, given its interaction with ethnic identity, gender, social class, and other dimensions of stratification.

Within Peruvian psychological research, the available evidence remains limited. Experiences of ethnoracial discrimination have been associated with depression, anxiety, and suicidal ideation through emotional responses such as anger, shame, and sadness (Sánchez-Villena and Temple-Focón, 2023). A systematic review found that Peruvian research on the psychological consequences of discrimination remains scarce and methodologically heterogeneous (Sánchez-Villena and Temple-Focón, 2025). Furthermore, a recent study found a small negative association between discrimination and self-esteem but no association with subjective well-being in a Peruvian sample, confirming that associations with psychological outcomes vary according to the dimension examined (Sánchez-Villena and Temple-Focón, 2026). These differences highlight the need to examine psychological well-being and psychological distress separately and to investigate the possible mechanisms linking phenotypic hierarchies to these outcomes.

Despite these advances, few studies have jointly examined skin tone, experiences of discrimination, and mental health among Peruvian university students. Existing research has primarily focused on general racial or ethnic discrimination, whereas skin color, understood as a continuous phenotypic marker, has received less attention. Distinguishing skin color from ethnoracial self-identification is important because individuals may receive different treatment according to their skin tone, even when they share the same ethnoracial identity (Telles et al., 2015). To address this gap, the present study aimed to examine the mediating role of experiences of discrimination in the associations between skin color and psychological well-being and distress among university students from Metropolitan Lima.

Based on the preceding evidence, the following hypotheses were proposed:

*Hypothesis 1*: Darker skin tones will be associated with lower levels of psychological well-being and higher levels of psychological distress among Peruvian university students.

*Hypothesis 2*: Darker skin tones will be associated with greater experiences of discrimination among Peruvian university students.

*Hypothesis 3*: Greater experiences of discrimination will be associated with lower levels of psychological well-being and higher levels of psychological distress among Peruvian university students.

*Hypothesis 4*: Experiences of discrimination will significantly mediate the associations between skin color and psychological well-being and psychological distress (Figure 1).

**Figure 1:**
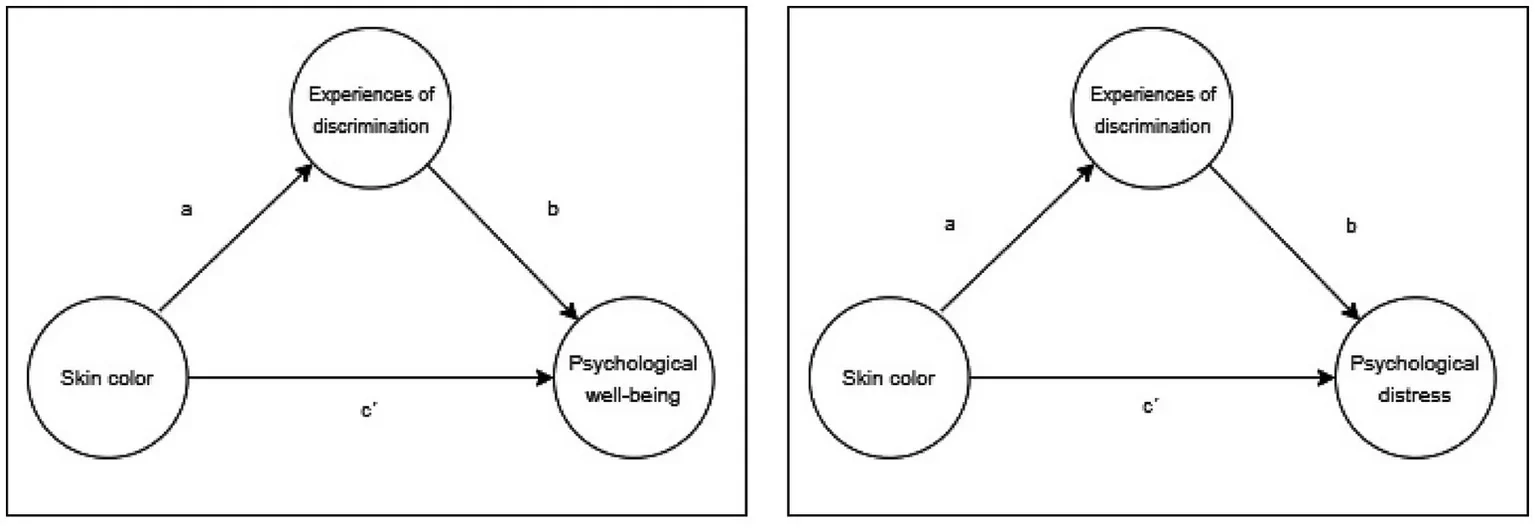
Hypothesized mediation models of experiences of discrimination in the association between skin color and psychological well-being (left panel) and psychological distress (right panel).

## Methods

### Design

A cross-sectional correlational design was used to examine the associations among skin color, experiences of discrimination, and mental health.

### Participants

A total of 603 university students from a private university in Metropolitan Lima were recruited. The inclusion criteria were as follows: (a) being male or female, (b) being 18 years of age or older, (c) being enrolled at a private university, and (d) voluntarily agreeing to participate in the study. A nonprobability convenience sampling method was used. Recruitment involved prior coordination with the participating university, after which the instruments were administered to students who met the eligibility criteria.

Participants ranged in age from 18 to 38 years (M = 21.4, SD = 4.8). Of the total sample, 53.9% were women and 46.1% were men. The largest proportion of students were enrolled in the second academic semester (25.5%). Regarding employment status, 54.7% were not employed, whereas 31.3% were employed. Most participants self-identified as Mestizo (72.8%), followed by Andean (11.6%). Full details of the sociodemographic characteristics are presented in Table 1.

**Table 1:** Sociodemographic characteristics of the sample (*n* = 603).

Variable	Category	*f*	%
Sex	Male	278	46.1
	Female	325	53.9
Academic semester	1st semester	42	7
	2nd semester	154	25.5
	3rd semester	29	4.8
	4th semester	88	14.6
	5th semester	43	7.1
	6th semester	91	15.1
	7th semester	42	7
	8th semester	80	13.3
	9th semester	16	2.7
	10th semester	18	3
Employment status	Employed	189	31.3
	Unemployed	330	54.7
	Pre-professional internship	84	13.9
Socioeconomic status	Low	27	4.5
	Lower-middle	123	20.4
	Middle	387	64.2
	Upper-middle	61	10.1
	High	5	0.8
Ethnic identity	Andean	70	11.6
	Afro-Peruvian	27	4.5
	Mestizo/a	439	72.8
	White	67	11.1
Born in Peru	Yes	593	98.3
	No	10	1.7
Place of origin	Lima	463	76.8
	Province	130	21.6
	Foreign country	10	1.7
Native language	Quechua	7	1.2
	Aymara	1	0.2
	Other native language	1	0.2
	Spanish	592	98.2
	Other foreign language	2	0.3
Type of housing	House	390	64.7
	Apartment	163	27
	Rented room	50	8.3
Living arrangement	Living alone	50	8.3
	With nuclear family (father and mother)	257	42.6
	With extended family (parents and other relatives)	211	35
	With friends or roommates	7	1.2
	With relatives (not parents)	78	12.9
Health insurance	Yes	457	75.8
	No	146	24.2

### Instruments

#### Sociodemographic questionnaire

The sociodemographic questionnaire collected information on participants’ age, sex, ethnic identity, academic semester, occupational status, socioeconomic status, place of birth, place of origin, native language, housing type, household composition, and health insurance status.

#### Experiences of discrimination

The Experiences of Discrimination (EOD) Scale was developed by Krieger et al. (2005) and comprises nine items designed to assess experiences of discrimination across different situations, such as while receiving healthcare or in public settings. Each item is rated on a scale ranging from 1 (never) to 4 (four or more times). This instrument was selected because a psychometric adaptation had been conducted in Peru by Sánchez-Villena and Temple-Focón (2023), who provided evidence of validity, reliability, and measurement invariance. They also linguistically adapted the EOD items to the Peruvian context and reported favorable model-fit and internal consistency indices (CFI = 0.97, SRMR = 0.06, ω = 0.80).

#### Skin color

An adapted version of the chromatic palette originally developed by the Project on Ethnicity and Race in Latin America (PERLA) was used. The original scale comprises 11 skin tones ordered from lightest to darkest. Before data collection, the palette was reviewed by five expert judges, who evaluated the cultural relevance and comprehensibility of its response options for the Peruvian population. The judges recommended using a 10-tone scale by removing the lightest tone. This modification was intended to reduce a possible tendency to select a skin tone lighter than the one perceived. The resulting adapted scale comprised 10 tones, with 1 representing the lightest tone and 10 the darkest.

Participants viewed a printed image of the scale and compared it with the inner surface of their forearm, selecting the tone they considered closest to their own skin color. The complete score ranging from 1 to 10 was retained in all inferential analyses. No cutoffs were established, and the scores were not grouped into categories.

#### Mental health

The five-item Mental Health Inventory (MHI-5) was originally developed by Veit and Ware (1983) for use in the general population (Berwick et al., 1991). The instrument comprises five items with four response options, originally coded from 1 (never) to 4 (always). For the analyses, responses were recorded from 0 to 3.

This measure was selected because Vilca et al. (2022) provided psychometric evidence supporting its use in the Peruvian context. Their findings supported a two-dimensional structure comprising psychological well-being (Items 2 and 4) and psychological distress (Items 1, 3, and 5). Confirmatory factor analysis produced favorable fit indices (CFI = 0.99, TLI = 0.99, RMSEA = 0.071), and acceptable internal consistency coefficients were reported for psychological well-being (ω = 0.75) and psychological distress (ω = 0.79).

### Procedures

The study was reviewed and approved by the Institutional Research Ethics Committee of Universidad Tecnológica del Perú (UTP; approval No. 265-2025-CEI-UTP). Before data collection, permission was coordinated with the academic authorities of the participating university and the instructors responsible for the selected courses. Institutional authorization was obtained, and the dates, times, and classrooms for administering the instruments were established according to the university’s availability.

On the scheduled assessment dates, a member of the research team entered the authorized classrooms. Before students were invited to participate, the instructor was asked to leave the classroom temporarily. This measure was intended to reduce any perceived pressure or undue influence associated with the instructor’s presence and to allow students to decide freely whether to participate. Students were invited directly during the researcher’s visits to the authorized classrooms. No recruitment flyers were used, and no incentives were offered for participation.

The study procedures, voluntary nature of participation, confidentiality of the information, and potential risks and benefits were explained verbally. Each student was then provided with a printed informed consent form. Students who agreed to participate and signed the consent form received the paper-and-pencil assessment protocols and a printed image of the chromatic scale used to identify their skin color. Data were collected between October and November 2025.

### Data analysis

First, the database was screened for missing values, floor effects, and outliers. Multivariate normality was assessed using Mardia’s coefficient, which is particularly appropriate for multivariate statistical procedures and factor analysis (Enomoto et al., 2020). Multivariate outliers were assessed using Mahalanobis distance (Groenewald and Van Vuuren, 2024). Descriptive analyses included frequencies and percentages for the sociodemographic variables and means, standard deviations, skewness and kurtosis coefficients, and minimum and maximum values for the study variables.

Evidence of the internal structure of the latent constructs was evaluated using confirmatory factor analysis. Given the ordinal nature of the items assessing experiences of discrimination and mental health, the models were estimated using the weighted least squares mean- and variance-adjusted estimator (WLSMV; DiStefano and Morgan, 2014). Standardized factor loadings greater than 0.40 were considered adequate, indicating that the observed variables accounted for a meaningful proportion of variance in the latent factor (Mueller and Hancock, 2015). Internal consistency reliability and convergent validity were assessed using McDonald’s omega (ω), composite reliability (CR), and average variance extracted (AVE).

Because skin color was treated as a 10-level ordinal variable scored from 1 to 10 and the distributions of experiences of discrimination and mental health deviated from normality, Spearman’s rank-order correlation coefficient was used (Hauke and Kossowski, 2011). Linear regression models were adjusted for sex, age, and socioeconomic status. HC3 robust standard errors were estimated because they demonstrated reliable performance under heteroscedasticity and nonnormality (Rajh-Weber et al., 2025).

Finally, statistical mediation models were tested, with experiences of discrimination specified as the mediator of the associations between skin color and mental health. Given the two-dimensional structure of mental health, separate mediation models were estimated for psychological well-being and psychological distress. Direct, indirect, and total effects were calculated. The 95% confidence intervals for the indirect effects were estimated using 5,000 bootstrap resamples. An indirect effect was considered statistically significant when its confidence interval did not include zero.

## Results

### Preliminary analysis

Data quality was assessed by examining compliance with the relevant statistical assumptions. Missingness was low, with less than 2% of responses missing per item and no evidence of systematic missing-data patterns. The items assessing experiences of discrimination showed a floor effect, with medians of 1 and responses predominantly concentrated in the lowest response category. Mental health indicators displayed moderate variability within the expected range of 0–3.

Multivariate normality was assessed using Mardia’s test, which revealed significant departures from multivariate normality in both skewness (*g*₁ = 27,683.77, *p* < 0.001) and kurtosis (*g*₂ = 145.89, *p* < 0.001). Univariate outliers were identified using standardized scores (|z| > 3.29), detecting 61 participants (10.1%) with at least one extreme value. Multivariate outliers were assessed using Mahalanobis distance (*p* < 0.001), identifying 47 cases (7.8%). A detailed inspection confirmed that all values fell within the theoretically permissible ranges and did not represent data-entry errors; therefore, no cases were excluded.

Given the ordinal nature of the indicators, the departures from multivariate normality, and the structural skewness of the discrimination items, the measurement models were estimated using the weighted least squares mean- and variance-adjusted estimator (WLSMV), which was considered more appropriate than maximum likelihood estimators under these conditions.

### Descriptive statistics

Table 2 presents the descriptive statistics for the study variables. Skin color was assessed using an ordinal chromatic scale ranging from 1 to 10 and had a mean score of 3.59 (SD = 0.93). The distribution showed moderate positive skewness (g₁ = 1.28) and elevated kurtosis (g₂ = 7.08), indicating a greater concentration of participants in the lighter-to-intermediate range of the scale. Experiences of discrimination had a low mean score (M = 1.24, SD = 0.37), with positive skewness (g₁ = 2.26) and elevated kurtosis (g₂ = 5.48). This distribution showed a pronounced floor effect, consistent with the low self-reported frequency of discriminatory experiences. Regarding mental health, psychological well-being had a mean score of 1.72 (SD = 0.64), whereas psychological distress had a mean score of 1.64 (SD = 0.67). In terms of distributional shape, psychological well-being showed an approximately symmetrical distribution (g₁ = 0.09), whereas psychological distress displayed slight negative skewness (g₁ = −0.31).

**Table 2:** Descriptive statistics.

Variable	M	SD	g1	g2	Min	Max
Skin color	3.59	0.93	1.28	7.08	1	10
Experiences of discrimination	1.24	0.37	2.26	5.48	1	4
Mental health						
Psychological well-being	1.72	0.64	0.09	−0.4	0	3
Psychological distress	1.64	0.67	−0.31	−0.39	0	3

M, Mean; SD, Standard deviation; g1, Skewness coefficient; g2, Kurtosis coefficient; Min, Minimum value; Max, Maximum value.

### Factor loadings and reliability

Factor loadings were of adequate magnitude and were statistically significant (*p* < 0.001) for most items, suggesting that the observed indicators adequately represented their respective latent factors. For experiences of discrimination, standardized factor loadings ranged from 0.689 to 0.811, and all items were statistically significant (*p* < 0.001), supporting a robust factor structure. Reliability was high (ω = 0.927, CR = 0.927), and the average variance extracted was 0.595, supporting the internal consistency and convergent validity of the measure. Regarding the two-dimensional structure of mental health, the psychological well-being subscale showed factor loadings ranging from 0.696 to 0.884. Its reliability coefficients were acceptable (ω = 0.698, CR = 0.698), and the AVE was 0.694, indicating that the latent construct accounted for an adequate proportion of variance in its indicators. The psychological distress dimension showed factor loadings ranging from 0.650 to 0.813, with adequate reliability indices (ω = 0.749, CR = 0.749) and evidence of convergent validity (AVE = 0.583). Overall, these findings indicate that the measures demonstrated satisfactory psychometric properties in the study sample (Table 3).

**Table 3:** Factor loadings of the confirmatory model.

Factor	Item	λ (Std.)	SE	*p*	ω	CR	AVE
Experiences of discrimination	ED01	0.689	0	—	0.927	0.927	0.595
	ED02	0.792	0.086	< 0.001			
	ED03	0.77	0.089	< 0.001			
	ED04	0.806	0.091	< 0.001			
	ED05	0.811	0.093	< 0.001			
	ED06	0.778	0.085	< 0.001			
	ED07	0.742	0.101	< 0.001			
	ED08	0.699	0.085	< 0.001			
	ED09	0.785	0.087	< 0.001			
Mental health: Psychological well-being	SM02	0.884	0.06	—	0.698	0.698	0.694
	SM04	0.696	0.062	< 0.001			
Mental health: Psychological distress	SM01	0.65	0	—	0.749	0.749	0.583
	SM03	0.813	0.085	< 0.001			
	SM05	0.746	0.072	< 0.001			

λ (Std.), standardized factor loading; SE, standard error; ω, McDonald’s omega; CR, composite reliability; AVE, average variance extracted. For model identification purposes, the first loading of each latent factor was fixed; therefore, no significance test was estimated for those parameters.

### Regression analyses

Table 4 presents the regression models adjusted for sex, age, and socioeconomic status, which were used to test Hypotheses 1, 2, and 3. Skin color was not significantly associated with psychological well-being (*B* = 0.004, SE = 0.032, *t* = 0.124, *p* = 0.902, *β* = 0.006) or psychological distress (*B* = 0.027, SE = 0.027, *t* = 0.989, *p* = 0.323, *β* = 0.037); therefore, Hypothesis 1 was not supported. In contrast, darker skin tones were associated with greater experiences of discrimination (*B* = 0.036, SE = 0.018, *t* = 2.025, *p* = 0.043, *β* = 0.089), supporting Hypothesis 2. Experiences of discrimination were also associated with lower psychological well-being (*B* = −0.191, SE = 0.078, *t* = −2.438, *p* = 0.015, *β* = −0.111) and greater psychological distress (*B* = 0.456, SE = 0.075, *t* = 6.080, *p* < 0.001, *β* = 0.256), supporting Hypothesis 3.

**Table 4:** Regression analysis.

Dependent variable	Predictor	*B*	*SE*	*t*	*p*	*β*
Experiences of discrimination	Skin color	0.036	0.018	2.025	0.043	0.089
Psychological well-being	Skin color	0.004	0.032	0.124	0.902	0.006
Psychological distress	Skin color	0.027	0.027	0.989	0.323	0.037
Psychological well-being	Experiences of discrimination	−0.191	0.078	−2.438	0.015	−0.111
Psychological distress	Experiences of discrimination	0.456	0.075	6.08	< 0.001	0.256

*B*, unstandardized coefficient; *SE*, robust standard error (HC3); *β*, standardized coefficien*t*.

### Mediation analysis

Table 5 presents the direct, indirect, and total effects of the mediation models examining the role of experiences of discrimination in the associations between skin color and mental health. The mediation analysis showed a positive and statistically significant indirect effect of skin color on psychological distress through experiences of discrimination (*B* = 0.016, 95% CI [0.001, 0.034]). Although the direct effect of skin color on psychological distress was not statistically significant, the findings were consistent with an indirect association through greater exposure to experiences of discrimination.

**Table 5:** Direct, indirect, and total effects of the mediation models.

Model paths	Estimated effect	SE	z	*p*	95% CI
Direct effect					
Skin color → Discrimination	0.036	0.017	2.088	0.037	0.003; 0.071
Skin color → Psychological well-being	0.011	0.032	0.345	0.73	−0.052; 0.072
Discrimination → Psychological well-being	−0.194	0.077	−2.506	0.012	−0.352; −0.047
Indirect effect					
Skin color → Discrimination → Psychological well-being	−0.007	0.005	−1.444	0.149	−0.019; 0.000
Total effect	0.004	0.032	0.126	0.9	−0.059; 0.065
Direct effect					
Skin color → Discrimination	0.036	0.017	2.088	0.037	0.003; 0.071
Skin color → Psychological distress	0.01	0.028	0.375	0.708	−0.047; 0.066
Discrimination → Psychological distress	0.454	0.076	5.986	< 0.001	0.303; 0.605
Indirect effect					
Skin color → Discrimination → Psychological distress	0.016	0.008	1.965	0.049	0.001; 0.034
Total effect	0.027	0.028	0.959	0.337	−0.031; 0.082

SE, standard error; CI, confidence interval. Indirect effects were estimated using bootstrap confidence intervals.

In contrast, the indirect effect of skin color on psychological well-being was not statistically significant (*B* = −0.007, 95% CI [−0.019, 0.000]). The corresponding direct and total effects are also reported in Table 5, and the mediation models are shown in Figure 2.

**Figure 2:**
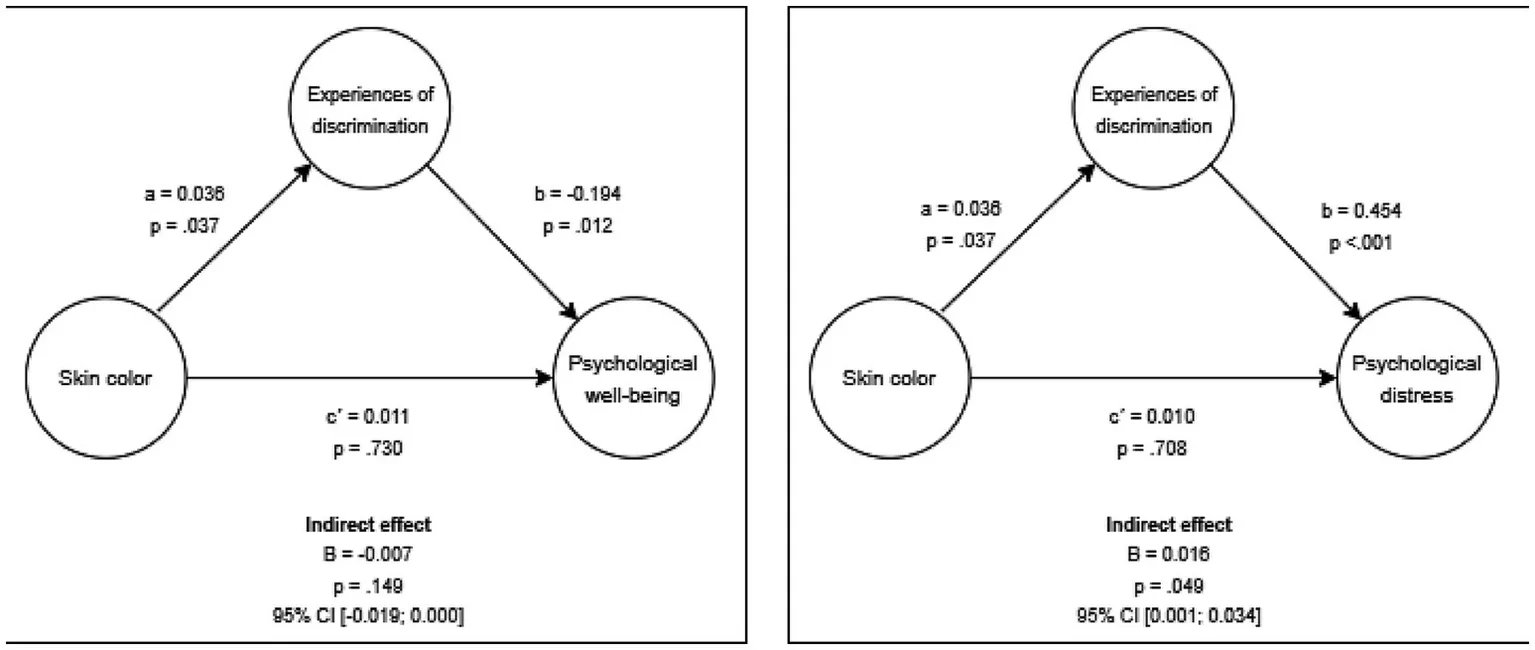
Mediation models examining the role of experiences of discrimination in the relationship between skin color and psychological well-being (left panel) and psychological distress (right panel).

## Discussion

The present study examined the associations among skin color, experiences of discrimination, and mental health among Peruvian university students. It also tested a statistical mediation model in which experiences of discrimination were examined as a mediator of the associations between skin color and psychological well-being and distress. Skin color was not directly associated with either dimension of mental health; therefore, Hypothesis 1 was not supported. In contrast, darker skin tones were associated with greater experiences of discrimination, which were, in turn, associated with lower psychological well-being and greater psychological distress, supporting Hypotheses 2 and 3. Finally, a significant indirect effect of skin color on psychological distress through experiences of discrimination was found, whereas the indirect effect on psychological well-being was not significant. Thus, Hypothesis 4 was partially supported.

Contrary to Hypothesis 1, skin color was not directly associated with psychological well-being or psychological distress in the university student sample. This finding suggests that skin tone was not immediately related to either dimension of mental health. Nevertheless, the absence of a direct association does not imply that skin color lacks psychological relevance. Rather, its consequences may depend on the social meanings, evaluations, and experiences associated with phenotypic hierarchies. Consistent with this interpretation, Williams et al. (2025) reported that skin tone was not significantly associated with psychological distress or the perceived need for mental health services among Black and Hispanic emerging adults. This finding suggests that the relevance of skin tone may vary across contexts and that skin tone alone may be insufficient to explain variability in mental health outcomes. Similarly, Hamler et al. (2022) noted that the literature on skin tone and mental health has produced mixed findings. Although they found no direct association between skin tone and mental health, they observed that its relevance emerged through conditional effects involving discrimination. Thus, the expected differences according to skin tone may not always be detected in statistical models, particularly when other social variables are involved (Confer and Kopf, 2026). Consequently, the psychological implications of skin color may not manifest directly or uniformly but may depend on the social contexts in which phenotypic characteristics are perceived and evaluated.

Hypothesis 2 was supported, as darker skin tones were associated with greater experiences of discrimination. Although the magnitude of this association was small, its direction was consistent with previous research. Skin color constitutes a socially visible phenotypic marker through which people may be perceived, categorized, and socially stratified (Craddock et al., 2025; Painter II et al., 2020; Patterson and Grob, 2026). Hunter (2016) argued that colorism operates as a subsystem of structural racism, increasing the frequency and severity of unequal treatment among racialized groups and affecting their educational and social trajectories. In Latin America, colonial stratification was organized around skin color and other phenotypic characteristics, whereas mestizaje and whitening practices contributed to obscuring the persistence of privileges associated with proximity to Whiteness (Chavez-Dueñas et al., 2014; Rejón, 2024). Similarly, Painter II et al. (2020) noted that Latin American pigmentocracies are characterized by the central role of skin tone as a source of inequality, even when it is distinguished from ethnoracial self-identification. For example, Marrow et al. (2022) found that darker-skinned Mexicans reported higher levels of discrimination, even after controlling for demographic, socioeconomic, and immigration-related factors. Harris (2022) also reported that Black and darker-skinned adolescents in Brazil and Colombia experienced greater perceived discrimination in educational contexts shaped by social inequality. This pattern has also received empirical support in Peru, where darker-skinned Afro-Peruvians had fewer opportunities to access higher education, even after accounting for their social backgrounds. This finding reinforces the role of skin color as a relevant dimension of stratification within Peruvian society (Benavides et al., 2019). In this sense, skin tone is not merely an individual physical characteristic but also a social marker that may increase exposure to unequal treatment.

Hypothesis 3 was supported because experiences of discrimination were associated with lower psychological well-being and greater psychological distress. These findings suggest that discrimination constitutes a proximal psychosocial stressor with important implications for mental health. Previous evidence has consistently shown that discrimination is associated with poorer mental health, particularly psychological distress, depressive symptoms, and anxiety (Emmer et al., 2024; Pichardo et al., 2021; Wang et al., 2026). Among Brazilian university students, Bastos et al. (2014) found that discrimination attributed to skin color, race, and social class was associated with a greater likelihood of common mental disorders. Similarly, Sánchez-Villena and Temple-Focón (2023) identified experiences of ethnoracial discrimination as social stressors with implications for mental health in the Peruvian population. Collectively, these findings position discriminatory treatment as a social experience that is more proximally associated with emotional harm than the phenotypic attribute around which such discrimination is organized.

The partial support for Hypothesis 4 provides a more specific explanation of how colorism may be associated with mental health. Although skin color was not directly associated with psychological distress, darker skin tones were related to greater exposure to experiences of discrimination, which were, in turn, associated with greater distress. This pattern frames colorism as a social and relational process rather than as a psychological consequence inherent to skin pigmentation. Skin tone functions as a visible marker around which social hierarchies and evaluations are organized, whereas unequal treatment constitutes a proximal mechanism through which these hierarchies may translate into emotional consequences. This interpretation is consistent with studies documenting discrimination as an intermediate process linking skin tone to different health outcomes (Landor et al., 2026; Louie, 2020; Monk, 2015). The finding that this indirect pathway emerged only for psychological distress and not for psychological well-being is also consistent with literature conceptualizing these domains as related but distinct dimensions of mental health. Moreover, previous research has generally documented more robust associations between discrimination and indicators of negative mental health than between discrimination and positive mental health outcomes.

The difference between the findings for psychological distress and psychological well-being provides insight into the specificity of the potential mental health consequences of colorism. Although these dimensions are related, they do not necessarily represent opposite ends of a single continuum. The two-continua model proposes that the presence of psychological distress and the experience of positive well-being constitute distinct dimensions of mental health that may respond to different psychosocial determinants (Mason Stephens et al., 2023; Westerhof and Keyes, 2010). Consistent with this distinction, Wang et al. (2026) found more consistent associations between discrimination and negative mental health indicators than between discrimination and positive indicators. Experiences of unequal treatment may therefore be more immediately associated with negative emotional responses, such as tension, sadness, or worry, whereas psychological well-being may also depend on broader protective resources, including social support, a sense of belonging, and coping strategies. Accordingly, the absence of a significant indirect effect on psychological well-being does not necessarily indicate that colorism is irrelevant to this dimension. Rather, in the present sample, the indirect association was more evident for the generation of psychological distress than for the reduction of positive psychological experiences.

Beyond extending the available evidence to an understudied Peruvian university context, the present study contributes to the colorism literature by distinguishing the phenotypic marker from the social process through which it may acquire psychological consequences. The findings suggest that skin color should not be conceptualized as an individual risk characteristic but as a socially interpreted attribute embedded within relationships and institutions that may reproduce phenotypic hierarchies. This distinction shifts the explanation away from individual pigmentation and toward practices of unequal treatment and the social contexts in which these practices occur. Universities should therefore address discrimination not only as an ethical issue but also as a matter related to student well-being and mental health. Higher education institutions should strengthen their procedures for preventing and reporting everyday discrimination, incorporate phenotypic hierarchies into awareness-raising initiatives, and consider these experiences within psychological support and student well-being services.

Although the findings provide relevant evidence, several limitations should be considered. First, the cross-sectional design precludes causal inferences regarding the associations among the study variables. Second, the sample was recruited using nonprobability convenience sampling; therefore, the findings cannot be generalized to the entire Peruvian university student population. Third, the concentration of participants in the lower and intermediate ranges of the chromatic scale may have restricted the variability required to identify stronger associations. Fourth, the floor effect observed in the discrimination measure may have attenuated the estimated associations. Fifth, the observed indirect effect was small, and its confidence interval was close to zero; therefore, it should be interpreted cautiously. Sixth, skin color was self-assessed by comparing the chromatic scale with the inner surface of the forearm. Although this procedure provided a standardized anatomical reference, this area is generally less visible during social interactions, and its pigmentation may differ from that of the face and other exposed body areas. Consequently, this measure may not fully capture socially perceived skin tone and may have influenced the estimated associations. In addition, the self-assessment of skin color and the self-reporting of experiences of discrimination may be affected by perceptual processes, recall bias, or social desirability. Future longitudinal and multicenter studies should include public and private universities from different regions of Peru, incorporate assessments of visible skin areas and observer-rated measures, and examine protective resources such as social support, sense of belonging, and coping strategies.

Overall, the present study provides evidence from a Peruvian university context in which skin color acquired psychosocial relevance primarily through its association with greater exposure to experiences of discrimination. This indirect pathway was more clearly evident for psychological distress than for psychological well-being. Although these findings were interpreted within the documented context of ethnoracial inequality in Peru, the study did not directly assess institutional or structural indicators of discrimination. Therefore, the results do not constitute direct evidence of structural racism but rather provide evidence of an interpersonal mechanism that is consistent with this broader social context.

## Data Availability

The datasets presented in this article are not publicly available because they contain potentially sensitive participant information concerning skin color, experiences of discrimination, mental health, and sociodemographic characteristics. De-identified data may be made available to qualified researchers upon reasonable request to the corresponding author, subject to applicable ethical and institutional requirements. Requests should be directed to Julio Dominguez-Vergara at C20928@utp.edu.pe.
