# Supplementary material for: Skin color and mental health in Peru: the mediating role of experiences of discrimination among university students
Source: Front Psychol. 2026 Sep 30;17:1867013. doi: 10.3389/fpsyg.2026.1867013 (PMC13661418; doi:10.3389/fpsyg.2026.1867013)
Supplement: Supplementary file 1 [file Supplementary_file_1.docx]

**Supplementary material: Spearman correlation matrix**

Zero-order correlations among the study variables are presented in Table S1. Overall, the direction of these associations was consistent with the results obtained from the regression models.

Table S1. Spearman correlations among the study variables

| **Variable** | **1** | **2** | **3** |
| --- | --- | --- | --- |
| 1. Skin color (Higher score = darker skin tone) | — |  |  |
| 2. Experiences of discrimination | 0.102* | — |  |
| 3. Psychological well-being | 0.014 | -0.147*** | — |
| 4. Psychological distress | -0.008 | 0.250*** | -0.491*** |
